# An evolutionary game approach for determination of the structural conflicts in signed networks

**DOI:** 10.1038/srep22022

**Published:** 2016-02-26

**Authors:** Shaolin Tan, Jinhu Lü

**Affiliations:** 1 College of Electrical and Information Engineering, Hunan University, Changsha 410082, China; 2 Institute of Systems Science, Academy of Mathematics and Systems Science, Chinese Academy of Sciences, Beijing 100190, China

## Abstract

Social or biochemical networks can often divide into two opposite alliances in response to structural conflicts between positive (friendly, activating) and negative (hostile, inhibiting) interactions. Yet, the underlying dynamics on how the opposite alliances are spontaneously formed to minimize the structural conflicts is still unclear. Here, we demonstrate that evolutionary game dynamics provides a felicitous possible tool to characterize the evolution and formation of alliances in signed networks. Indeed, an evolutionary game dynamics on signed networks is proposed such that each node can adaptively adjust its choice of alliances to maximize its own fitness, which yet leads to a minimization of the structural conflicts in the entire network. Numerical experiments show that the evolutionary game approach is universally efficient in quality and speed to find optimal solutions for all undirected or directed, unweighted or weighted signed networks. Moreover, the evolutionary game approach is inherently distributed. These characteristics thus suggest the evolutionary game dynamic approach as a feasible and effective tool for determining the structural conflicts in large-scale on-line signed networks.

Signed networks are graphs with positive and negative edges to embody additional information about the relationships between members. The notion comes from a variety of studies such as social psychology and biological systems^1^,^2^,^3^,^4^,^5^,^6^,^7^,^8^,^9^,^10^,^11^. Generally, the positive/negative ties in signed networks can be used to characterize the friendly/hostile or cooperative/competitive relationships in social networks^1^,^2^,^3^,^4^,^5^, and are also suitable for representation of the activating/inhibiting interactions in biochemical networks^6^,^7^,^8^,^9^,^10^,^11^. In recent years, increasing interests have arisen to infer the dynamical behaviors of a social or biological network through analysis of the corresponding signed network^12^,^13^,^14^,^15^,^16^,^17^.

A core and extensively studied property of signed networks is structural balance. In social networks, structural balance, also called social balance, is formulated to understand the stability or tensions in population systems^18^,^19^,^20^,^21^. In biochemical networks, structural balance, equivalent to the monotonicity property, equips the dynamical system with useful properties as diverse as convergence, high predictability, and robustness^6^,^7^,^8^,^9^. Formally, a signed network is structurally balanced if and only if all its cycles have an even number of negative edges.

Real social and biological networks are usually not exactly balanced yet they are very near to balance^22^. In most cases, sign change of a small proportion of edges is sufficient to make real networks balanced. With regard to practical applications, two problems are often encountered in analysis of signed networks: Firstly, is the signed network balanced? Secondly, if not, at least how many (and which) edges should change sign or be pruned to make the network balanced? Here, the minimal numbers of edges, which should be deleted to make the network balanced, are called structural conflicts of the signed network. Indeed, the first problem is simple and has been completely solved by Heider^23^,^24^. Yet, the second problem turns out to be NP-hard from a computational perspective^25^.

At present, several heuristic methods have been proposed to determine the structural conflicts based on semi-definite programming^24^ or equivalence transformations to signed networks^9^,^22^,^26^,^27^,^28^. In spite of bringing significant achievements, these methods, all utilize a centralized optimizer, which needs complete information of the signed network each step. Yet, in the face of large-scale, time-varying, weighted, or directed signed networks—which are a result of the development of social media and system biology—centralized approaches become inefficient or even unworkable. As a consequence, distributed heuristic approaches, which allow agents themselves to optimize their own performances, becomes of utmost significance, especially for determining the structural conflicts in the future research.

In this paper, inspired by the intrinsic relationship between natural evolution and optimization^29^, we initiate a novel distributed heuristic approach to determine the structural conflicts in signed networks based on the spatial evolutionary game dynamics^30^,^31^,^32^,^33^,^34^. In the proposed approach, network nodes (agents) adaptively update their own choice of alliances to gain better performance without considering the impact on the whole network. Under the above microscopic self-adaption, the overall signed network will spontaneously divide into two stable alliances where the structural conflicts could be directly determined. This effective and easy method can also be applied to directed or weighted signed networks. Moreover, since the method is distributed, its computational time can be greatly reduced, which plays an important role in applications to large-scale on-line signed social networks.

## Results

### Signed networks and structural conflicts

**Figure 1:**
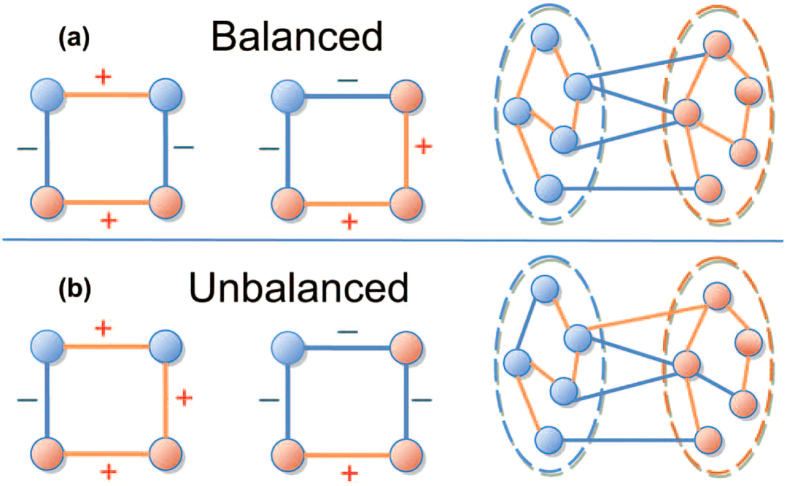
Illustration of signed networks. (**a**) Balanced graphs, and (**b**) Unbalanced graphs. In each graph, the colors of the nodes denote their alliances, and the colors of the edges denote the positive and negative signs, respectively.

A signed network can be denoted by a graph *G* = (*V, E*) with *V* = {*v*_*1*_*, v*_*2*_*, …, v*_*N*_} representing the nodes and *E* = {*e*_*ij*_*|i, j* = 1, 2*,…, N*} the edges, where *e*_*ij*_ = 1, −1, or 0 indicate a positive edge, a negative edge, or no edge between nodes *v*_*i*_ and *v*_*j*_, respectively (see Fig. 1). In most cases, since the relationship between individuals is mutual, the edges are assumed undirected, that is, *e*_*ij*_ = *e*_*ji*_. For convenience, hereafter let *N*_*i*_ = {*j|e*_*ij*_ ≠ *0, j* = *1, 2, …, N*} denote the neighboring nodes of node *v*_*i*_.

In balanced signed networks, the nodes can be divided into two opposite alliances such that the edges within each alliance are positive while those between two alliances are negative (see Fig. 1(a)). From a mathematical point of view, there exists a spin assignment *v*_*i*_ = 1 or −1 to each node *v*_*i*_ such that *v*_*i*_*e*_*ij*_*v*_*j*_ = 1 holds for all edges in balanced networks. However, in unbalanced networks, no matter how the nodes are divided, there always exist conflict edges, i.e. negative edges within the same alliance or positive edges between the opposite alliances (see Fig. 1(b)). Determining the structural conflicts means finding an optimal division of the nodes such that the number of conflict edges is minimal.

### The evolutionary game approach

Note that the above optimization problem is equivalent to assigning *v*_*i*_ = 1 or −1 to each node *v*_*i*_ so as to maximize


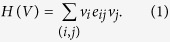


Denote the above function *H*(*V*) the fitness of the network. Here, we design an evolutionary game^35^,^36^,^37^,^38^ (see Supplementary Information (SI) for details) on the signed network *G* = (*V, E*) such that the nodes automatically adjust their strategies towards maximization of the above fitness function during the evolutionary process.

In detail, let each node *v*_*i*_choose a strategy from {1, −1}, representing the alliance it belongs to. Every node interacts in a game with all its neighbors as follows. For each pair of nodes, if they possess the same strategy and the relationship between them is positive, or they possess two opposite strategies and the relationship between them is negative, then both nodes obtain a positive unit payoff. Otherwise, if they possess the same strategy yet the relationship between them is negative, or they possess two opposite strategies and the relationship between them is positive, then both nodes obtain a negative unit payoff. Thus, the fitness (payoff) of each node is determined by


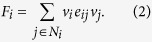


In the microscopic dynamics of the evolutionary process, nodes update their strategies according to the fitness landscape. We consider an aspiration-exploration updating dynamics as follows. Let the aspiration-level be zero. For a node *v*_*i*_, if its fitness is less than the aspiration level at time *t*, that is 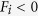, then the node abandons the present strategy and turns to adopt an opposite strategy at the next step. That is, *v*_*i*_(*t* + 1) = *−v*_*i*_(*t*). However, if the fitness of a node is equal to or greater than the aspiration level, that is 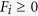, then the node explores other strategies with a designed probability *R*_*i*_. In detail, with probability *R*_*i*_, the node adopts a random strategy at the next step, otherwise, the node keeps to the previous strategy.

**Figure 2:**
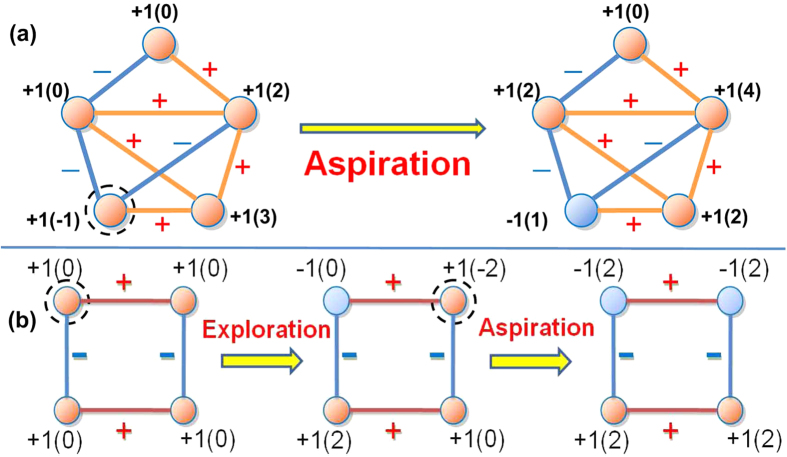
Illustration of optimization based on the evolutionary game dynamics. (**a**) Through the aspiration dynamics, nodes adjust their strategies to gain larger fitness, leading to better solutions to the optimization problem; (**b**) Exploration behaviors introduce new feasible solutions and help to overcome those local optimal solutions to the optimization problem. In each graph, 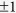 denotes the strategy of each node, and the values in the parentheses denote the fitness of each node.

In the above evolutionary process, the exploration behavior searches the solution space by bringing various new solutions, while the aspiration dynamics keeps better solutions and eliminates disadvantageous ones. As such, the evolutionary process leads to better performance of each node (see Fig. 2) in the long run. In practical applications, the exploration probability is set to be


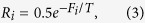


where 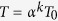 denotes the noise parameter. Here, 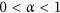 is the damping coefficient, *T*_*0*_ > 0 is the initial temperature, and 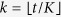, where *t* is the iteration step indicator, *K* is the damping period, and the notion  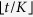 denotes the maximal integer less than *t/K*. (see SI for details about setting of 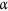, *T*_*0*_, and *K*). The above setting of exploration probability can help the network overcome those local fitness peaks and eventually reach to nearly maximal global fitness.

Note that the fitness of the whole network is just the summation of the fitness of each node. If the fitness of a node increases by *1*, then the fitness of the whole network will increase by *2*. Thus, the non-cooperative updating of each node could drive the signed network towards better fitness. Based on the above evolutionary game approach, a novel heuristic optimization algorithm is proposed (for more details, please refer to Methods). In what follows, we apply the proposed algorithm to different kinds of signed networks and illustrate its effectiveness and advantages.

### Determining the structural conflicts in undirected unweighted signed networks

To begin with, we test the performance of the evolutionary game approach with four large scale real-world signed networks, including the yeast (gene regulatory) network^39^, EGFR (epidermal growth factor receptor) pathway network^40^, macrophage (molecular interaction) network^41^, and *E.coli* (gene regulatory) network^42^. The numbers of nodes and edges of the above networks span from hundreds to thousands. Since all the above original networks contains a number of symmetric- incompatible and sign-ambiguous edges pairs, following previous practices^9^, all the above networks have been rendered undirected beforehand by deleting those incompatible edges pairs.

**Figure 3:**
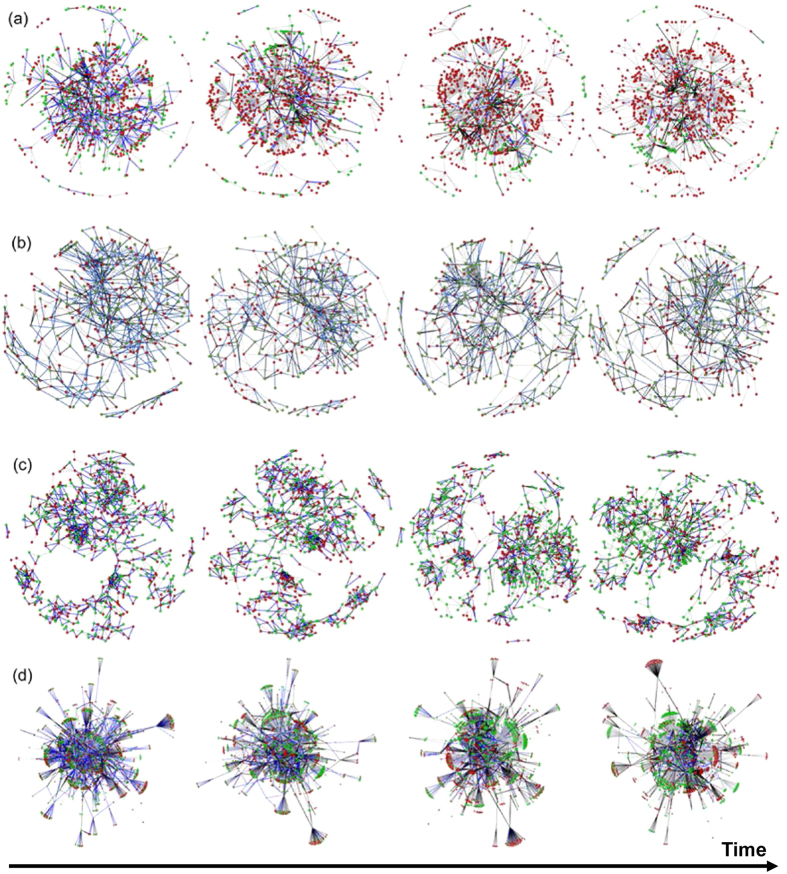
The evolution of alliances and structural conflicts in different signed networks under the evolutionary game dynamics. (**a**) The yeast network; (**b**) the EGFR pathway network; (**c**) the macrophage network; (**d**) the *E.coli* network. In the above networks, the red and green nodes denote two hostile alliances. The gray, black, and blue edges denote the positive, negative, and conflict edges, respectively. Under the evolutionary game dynamics, the nodes form two alliances spontaneously such that the number of conflict edges (i.e. the blue edges) in the signed networks decreases. The change of node locations from image to image is due to randomness of the network layout algorithm.

After imposing the proposed evolutionary game dynamics on the above signed networks, we find that all the nodes adaptively adjust their strategies (alliances) to gain better fitness. Moreover, though the strategy-updating process of each node is non-cooperative and self-interested, the number of conflict edges among the entire network decreases rapidly with strategy adjustment of nodes (see Fig. 3). Take the *E.coli* network as an example. The number of conflict edges among the alliances is 1195 at step *t* = 5000, whereas it decreases to 684 at step *t* = 15000, 484 at step *t* = 25000, and 376 at step *t* = 35000 (see Fig. 3(d)).

**Figure 4:**
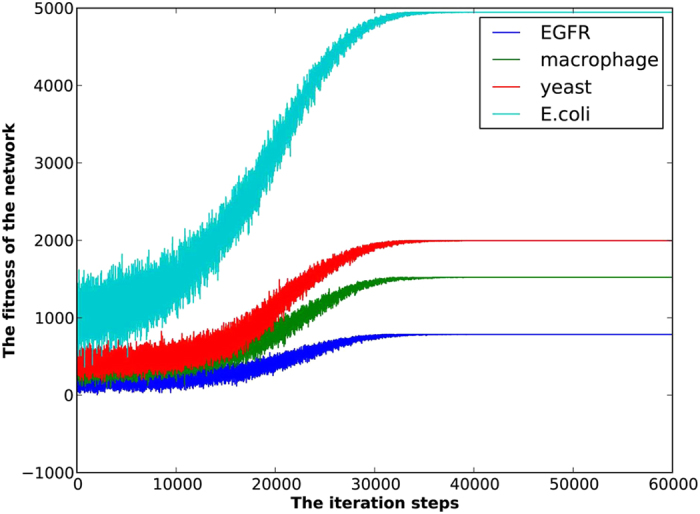
The evolutionary trajectory of the fitness of each signed network with the evolutionary game dynamics. Initially, all the nodes belong to the same alliances. Hence, the fitness of network is very low. The aspiration process promotes the fitness of entire network. While exploration behavior breaks local fitness peaks and brings in fluctuation to the fitness of network.

To gain a clear display of the optimization process with the evolutionary game approach, we record the evolutionary trajectory of the fitness of each network (see Fig. 4). Note that the number of conflict edges can be determined by the network size *|E|* and the network fitness *H*(*V*). In detail, if the number of structural conflicts is zero, then we have *H*(*v*) = 2*|E|.* Moreover, every increase of the number of structural conflicts leads to a decrease by 4 on the network fitness. Thus, the number of structural conflicts *s* can be derived as follows:


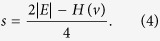


Since the exploration probability decreases to zero with time and the fitness of each node never decreases under the aspiration dynamics, the evolutionary game process of the signed networks will always converge to a stable equilibrium with nearly minimal structural conflicts. Table 1 displays the obtained minimal number of structural conflicts of the above four real-world signed networks. The results outperform those of refs 24 and 27 and equal to the best performance of a recent work^9^.

### Evaluating the performance of the evolutionary game approach

The evolutionary game approach can adaptively minimize the conflict edges in signed networks, yet it is still a doubt how close the obtained number of conflict edges is to the optimal solution. To address this problem, the performance of our method is further evaluated with a sequence of signed networks with a designated number of structural conflicts.

The designated signed networks are generated with the following method. Suppose the network contains *2N* nodes, 2*M*_*1*_positive edges, and *M*_*2*_ negative edges, and the designated number of structural conflicts is *s*. Firstly, divide the nodes into two equal parts and randomly connect the node pairs within each part with *M*_*1*_ positive edges. Then, randomly connect the node pairs between the two parts with *M*_*2*_ negative edges. Finally, randomly sample *s* edges from the edge set and reverse their signs.

**Figure 5:**
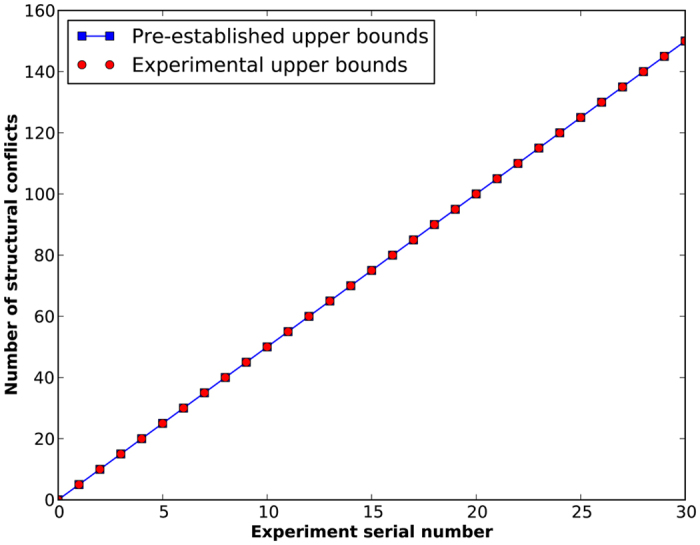
Comparison between the experimental and pre-designated upper bounds of the number of structural conflicts in designated signed networks. 31 experiments have been done and the designated number of structural conflicts uniformly distributed from 0 to 150.

Generally, the actual number of structural conflicts in the above signed network is equal to or slightly smaller than the designated number. Through the evolutionary game approach, it can be found that the obtained number of structural conflicts is also always equal to or smaller than the pre-established one in all the designated signed networks (see Fig. 5). This indicates that the structural conflicts in signed networks can be nearly accurately figured out by our method. The experimental results clearly validate the effectiveness of the evolutionary game approach.

### Determining the structural conflicts in weighted signed networks

In some social or biochemical networks, the intensity of friendly/hostile or activating/inhibiting relationships may vary from each other. In these cases, weighted signed networks are more proper to characterize the real-world systems^43^. In the following, we turn to address the problem of determining the structural conflicts in weighted signed networks.

Denote *G* = (*V, E, W*) a weighted signed network. Here, *W* = (*w*_*ij*_)^*N×N*^ is a weighted matrix, where each weight *w*_*ij*_ = *w*_*ji*_ can be positive, negative, or zero. Intuitively, the cost of changing the sign of a relationship with small weight is also small. Thus, to make a signed network balanced, it is more effective to change the sign of those insignificant edges. In this perspective, the structural conflicts of a weighted signed network are defined by a set of edges with minimal total weights, which should be deleted to make the network balanced. Correspondingly, the problem of determining the structural conflicts in weighted signed networks becomes to assign 1 or −1 to each node *v*_*i*_ such that the following function is maximized.


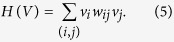


**Figure 6:**
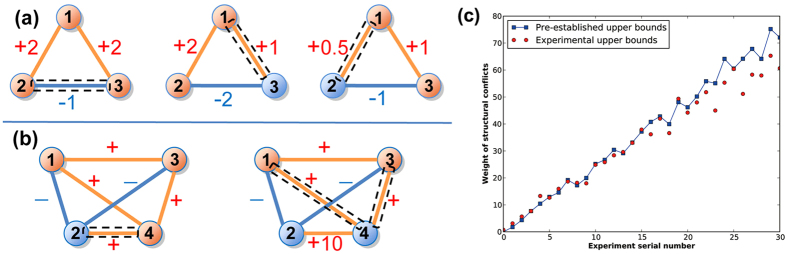
Structural conflicts in weighted signed networks. (**a**) If the network is unweighted, changing the sign of any edge will make the network balanced. However, if the network is weighted, the choice of the structural conflict is further determined by the weight of edges. (**b**) If the network is unweighted, it is optimal that node *v*_*1*_, *v*_*3*_, and *v*_*4*_ form an alliance and node *v*_*2*_ forms the other. In this case, *e*_*24*_ is the only structural conflict. However, if node *v*_*2*_ and *v*_*4*_ are very close friends, then it is more likely that node *v*_*2*_ and *v*_*4*_ form an alliance and node *v*_*1*_ and *v*_*3*_ form the other. That is, *e*_*14*_ and *e*_*34*_ are the structural conflicts. (**c**) Comparison between the experimental and pre-designated upper bounds of the total weight of structural conflicts in designated weighted signed networks.

Note that the definition of structural conflicts in weighted signed networks is a little different from that in unweighted signed networks. The assignment of weight to edges could greatly alter the choices of structural conflicts in the signed networks (see Fig. 6 (a,b)).

By redefining the fitness of each node as


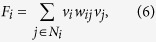


the above proposed evolutionary game approach can be directly applied to determine the structural conflicts in weighted signed networks without further modification. To explore the effectiveness of the evolutionary game approach, we initialize a sequence of weighted signed networks as testing samples, in which the tight upper bounds of structural conflicts are pre-established. The sequence of experimental weighted signed networks are generated with the same method as the experimental unweighted signed networks except that each edge is assigned with a random absolute weighted from (0, 1). In all the experiments, it can be found that the obtained upper bounds of structural conflicts agree with the pre-established ones excellently (see Fig. 6 (c)). Notably, the evolutionary game approach can also successfully determine the structural conflicts in weighted signed networks.

### Determining the structural conflicts in directed signed networks

**Figure 7:**
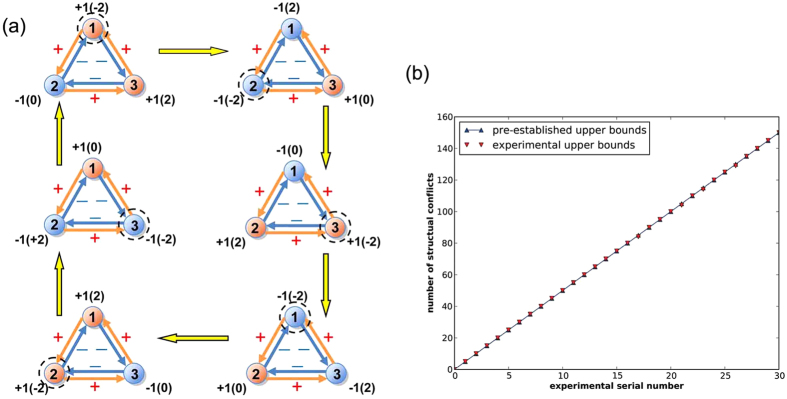
(**a**) Illustration of periodic evolution of the evolutionary game dynamics on directed signed networks. In the signed network, node *v*_*1*_ likes node *v*_*2*_, yet node *v*_*2*_ dislikes node *v*_*1*_. The same thing also happens between node pairs (*v*_*2*_, *v*_*3*_) and (*v*_*3*_, *v*_*1*_). The above situation can be encountered in some social networks. In this network, the fitness of the whole network is always zero. Thus, increase of the fitness of one node leads to decrease of the fitness of another, which results in a periodic evolution. In each graph, 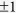 is the strategy of the nodes and the values in the parentheses denote the fitness of each node. (**b**) Comparison between the experimental and pre-designated upper bounds of the total number of structural conflicts in designated directed signed networks.

Directed signed networks are often encountered in social and biochemical networks^44^. In previous works, directed signed networks are commonly rendered into undirected networks for simplicity. Since the interactions in directed signed networks may be incompatible (e.g. 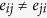), increase of the fitness of a node may not promise increase of the fitness of the entire network. In particular, in some cases, the proposed evolutionary game dynamics on directed signed networks may not reach consensus. Instead, it absorbs into a periodic orbit (see Fig. 7 (a)).

To solve this problem, we redefine the fitness of each node by


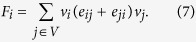


Numerical experiments show that the evolutionary game approach with the above modified fitness can effectively determine the number of directed structural conflicts in directed signed networks (see Fig. 7 (b)). As such, the evolutionary game approach may be seen as a universally applicable method for determining the structural conflicts in directed weighted signed networks.

## Discussion

The above results have shown the validity and effectiveness of the evolutionary game approach in determining the structural conflicts in unweighted or weighted, undirected or directed signed networks. The success of the evolutionary game approach is mainly driven by two factors. First, the aspiration dynamics leads to adaption of nodes. In the strategy updating process, each node adjusts its strategy to adapt to its neighbors in the network. And the self-adaption of each node drives the entire network towards better balance eventually. Note that the assignment of node fitness promises cooperative improvement of macroscopic fitness via self-interested adaption of microscopic updating.

Exploration behavior is the second key factor leading to the success of the evolutionary game approach. The entire network can often be trapped in stable states with local maximal fitness during the strategy updating process. Exploration behavior could constantly introduce various new feasible strategies into the network, which helps the network jump out of those local peaks and start evolving towards higher fitness landscapes. In particular, the gradual reduction of the exploration probability is of key importance in the optimization process. In detail, the fitness of nodes is very low initially. In this case, large exploration probability can rapidly drive the nodes to adopt more advantageous strategies. With the increase of fitness, the exploration probability decreases eventually. Finally, the exploration probability tends to zero and in this stage the entire network stabilizes at a state with nearly maximal fitness. The whole setting is similar to that in the simulated annealing (SA) algorithms^45^ (see SI for details).

It is noted that the proposed evolutionary game dynamics well characterizes the underlying formation process of alliances in real-world signed networks. For example, during the evolution of social alliances, an agent may alter its alliances due to the social tension from the opposite alliance. This process can be modeled by the aspiration rule in the evolutionary game dynamics. In addition, agents may also sometimes explore other possible strategies randomly to improve its situation. Correspondingly, this kind of behavior can be mimicked by the exploration process.

In summary, we have proposed an evolutionary game approach to determine the structural conflicts in signed networks. The proposed approach is effective for both unweighted/weighted and undirected/directed signed networks. Moreover, it is a distributed algorithm. That is, each node optimizes the performance of itself only with information of its neighbors. Moreover, through adaption among nodes, the structural conflicts in the networks can be nearly successfully determined. Signed networks underlying current social or biochemical systems are often diversified in different aspects. The connection patterns can be unweighted or weighted, and undirected or directed. The number of nodes may span from hundreds to hundreds of thousands. Moreover, the structure of the networks may be time-varying. In all these applications, the distributed evolutionary game approach provides an effective tool for exploring the formation process of alliances and determining the structural conflicts.

## Methods

### Algorithm

Our algorithm is based on the evolutionary game dynamics on complex networks. Generally, it consists of three parts: the initialization process, the one-step node updating process, and the termination process. Let strategy +1 and −1 represent the alliance the node belongs to. The detailed operations in each process are summarized as follows:

Input: A signed network *G* = (*V, E*), the damping coefficient 0 < *a* < *1,* initial temperature *T*_*0*_ *>* *0,* damping period K > *0,* and a specified minimum threshold of the exploration probability *R.*

S1. The initialization process: Given a signed network, assign all nodes to strategy +1 and the payoff of each node according to Eq. (2). Let the step indicator *t* = *0.*

S2. The one-step node updating process: In an arbitrary order, update the strategy of each node in turn. And after updating the strategy of a node, renew the payoff of the node and all its neighbors subsequently.

S2.1. Strategy updating: For each node, if its fitness is less than zero, then assign the node to an opposite strategy; otherwise, if its fitness is equal or greater than zero, then assign the node to a random strategy with a probability *R*_*i*_, given by Eq. (3).

S2.2. Payoff updating: Given that the strategy of the updating node has been changed, reverse the payoff of this node and add the payoff of its neighbors *v*_*j*_ by *2s*_*i*_*e*_*ij*_*s*_*j*_.

After all nodes have been given a chance to update its strategy, record the total fitness of the current network and add the step indicator *t* by 1.

S3. The termination process: if all the exploration probabilities are less than the specified minimum threshold *R*, then end the program; otherwise, turn to S2.

The specific setting of the exploration probability and other parameters is shown in the supplementary materials. Since the exploration rate decreases to zero with time, the program will always end in finite iterations. Moreover, the total number of one-step node updating processes does not depend on the size of the network; it is a constant which depends only on the parameters of the exploration probability. Note that in each one-step node updating process, the strategy and payoff of a node *v*_*i*_ update at most one and |*N*_*i*_| + 1 times, respectively. Here, |*N*_*i*_| refers to the number of neighbors of node *v*_*i*_. Thus, the computation complexity of the algorithm is linear increasing with the product of the number of nodes and edges in the signed network, which is polynomial time.

## Additional Information

**How to cite this article**: Tan, S. and Lü, J. An evolutionary game approach for determination of the structural conflicts in signed networks. *Sci. Rep.* **6**, 22022; doi: 10.1038/srep22022 (2016).

## Supplementary Material

Supplementary Information

## Figures and Tables

**Table 1:** The number of nodes |*V*|, the number of edges |*E*|, the final fitness *H*(*V*), and the obtained number of structural conflicts *s* of four kinds of real-world signed networks.

Networks	|*V*|	|*E*|	*H(V)*	*s*
Yeast	690	1080	1996	41
EGFR	329	779	786	193
Macrophage	678	1425	1522	332
E.coli	1461	3215	4946	371
