# Supplementary material for: An evolutionary game approach for determination of the structural conflicts in signed networks
Source: Sci Rep. 2016 Feb 26;6:22022. doi: 10.1038/srep22022 (PMC4768106; doi:10.1038/srep22022)
Supplement: Supplementary Information [file srep22022-s1.pdf]

An evolutionary game approach for determination of the structural conflicts in signed networks

Supplement Information

Shaolin Tan[1] & Jinhu Lü[2*]

1. College of Electrical and Information Engineering, Hunan University, Changsha 410082, China; 2. Institute of Systems Science, Academy of Mathematics and Systems Science, Chinese Academy of Sciences, Beijing 100190, China

Evolutionary game dynamics on complex networks. Evolutionary game dynamics on complex networks consists of three fundamental elements: 1) a network; 2) a game; and 3) a strategy updating rule [1], [2].

The network characterizes the interaction structure of components in the evolving systems. Generally, a graph $G = (V, E, W)$ is used to denote the interaction network, where $V = \{v_1, v_2, \cdots, c_N\}$, E, and $W = (w_{ij})_{N\times N}$ are the node set, edge set, and the weight matrix of the graph, respectively. In this work, the interaction structure of the addressed evolving system is a signed network. That is, the edge weight w_{ij} can either be positive or negative.

The game captures the interplay between components. Each node possesses a strategy and play games with all its neighbors. A payoff is obtained after the game interactions. Considering that the relationships of node pairs are homogeneous in most cases, it is usually assumed that all pairs of nodes play a unified game in previous works [3], [4]. Yet, in this work, we consider two different games in the networks. The nodes choose a strategy from $\{+1, -1\}$. If the relationship between two nodes is positive, then the following game is played:

	$+1$	-1
$+1$	1	-1
-1	-1	1

. (1)

And if the relationship between two nodes is negative, then an opposite game is played:

	$+1$	-1
$+1$	-1	1
-1	1	-1

. (2)

Thus, according to the above game interactions, the payoff of each node is

$$F_i = \sum_{v_j \in V} v_i w_{ij} v_j \tag{3}$$

That is, the node payoff depends not only on the strategies of its neighbors but also on the relationships with its neighbors.

The strategy updating rule describes the microscopic evolving process of the system. Each agent updates its strategy based on the information of the payoff and strategies of its neighbors and itself. Different kinds of updating rules have been introduced. In this work, we consider a selection-mutation rule: if the fitness of a node is less than a threshold, then selection happens; otherwise, if the fitness of a node is larger or equal than the threshold, then mutation happens with some rate. Let $v_i(t)$, $F_i(t)$, and $R_i(t)$ denote the strategy, payoff, and mutation rate of node v_i at time t. Let $\Theta = 0$ denote the threshold. Then, the selection-mutation rule can be written as follows:

$$v_i(t+1) = \begin{cases} v_i(t) & \text{with probability } R_i(t)/2 \text{ if } F_i(t) \geq \Theta, \\ -v_i(t) & \text{otherwise.} \end{cases} \tag{4}$$

Mutation rate. The value of mutation rate can greatly influence the pace and trajectory of the evolution process. Appropriate mutation rate can lead to a better performance. In this work, the mutation rate is set to be

$$R_i = 0.5e^{-F_i/T}, \tag{5}$$

where the noise parameter $T = \alpha^k T_0$. Here, $T_0 > 0$ is the initial noise parameter and $0 < \alpha < 1$ is the decaying exponential. Moreover, $k = \lfloor t/K \rfloor$, where t is the iteration steps, K is the damping period, and the notion $\lfloor t/K \rfloor$ denotes the maximal integer less than t/K.

The above setting of mutation rate has two novel features. First, the mutation rate depends on the node fitness. Nodes with larger fitness possess a smaller mutation rate, while those with smaller fitness possess a larger mutation rate. Such a setting is intuitively reasonable. Second, the mutation rate is eventually decreasing with time. The noise parameter is large initially and decreases to zero eventually, which is very similar to simulated annealing algorithms [5]. As a result, Eq. 5 is referred to heterogeneous decaying mutation rate in the following.

For comparisons, consider two other mutation rates:

(i) Constant mutation rate $R_i = R$. The mutation rate keeps unchanged during the evolutionary process. Moreover, it is the same for all nodes.

(ii) Heterogeneous mutation rate $R_i = 0.5e^{-F_i/T_0}$. The mutation rate only depends on the node fitness.

In the following, we explore the effect of the above settings of mutation rate on the evolutionary process in the yeast (gene regulatory) network [6].

Fig. S1 shows the evolutionary trajectories of the network fitness with different constant mutation rates. Since the mutation rate is a small constant, the network fitness can rapidly increase to a stable value. Yet, after that, the network fitness fluctuates around the stable value due to the non-zero mutation rate. Moreover, compared with that in the heterogeneous decaying mutation, the network fitness in constant mutation is much lower.

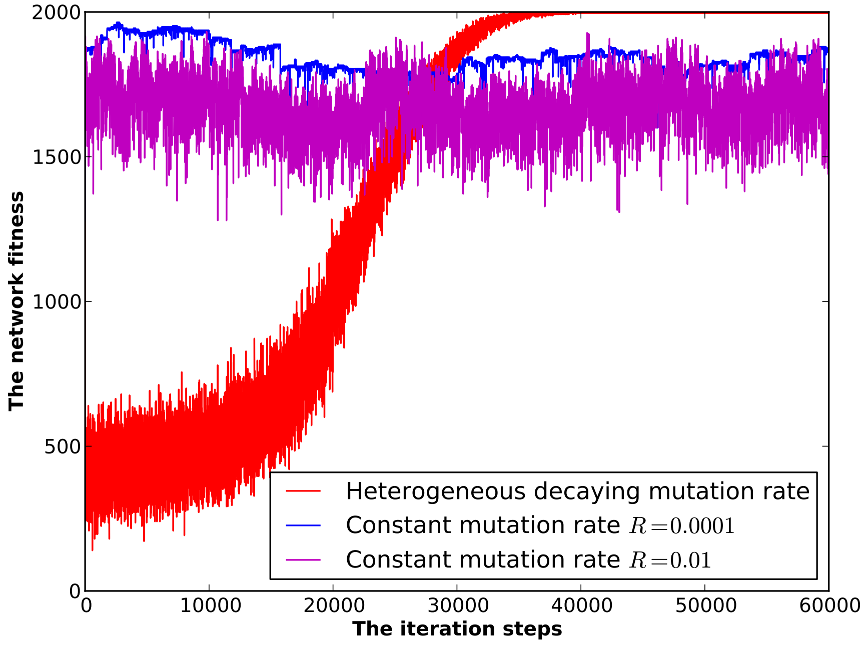

Fig. S1. Comparison of the evolutionary trajectories of the network fitness with different constant mutation rates and the heterogeneous decaying mutation rate. The initial noise parameter, decaying exponential, and damping period of the heterogeneous mutation rate are $T_0 = 100$, $\alpha = 0.9$, and $K = 100$, respectively.

Fig. S2 shows the evolutionary trajectories of the number of structural conflicts with different heterogeneous mutation rates. In the heterogeneous mutation rate, the noise parameter T is fixed. If the noise is large, then the evolutionary trajectory will fluctuate. However, if the noise is too small, then the evolutionary trajectory will end with a local optimal stable states with more structural conflicts. Thus, compared with the heterogeneous decaying mutation rate, the problem of the heterogeneous mutation rate lies in the difficulties in determination of the noise parameter.

Assignments of the initial noise parameter, decaying exponential, and damping period can also greatly affect the performance of the evolutionary game dynamic approach. Figure S3 shows the evolutionary

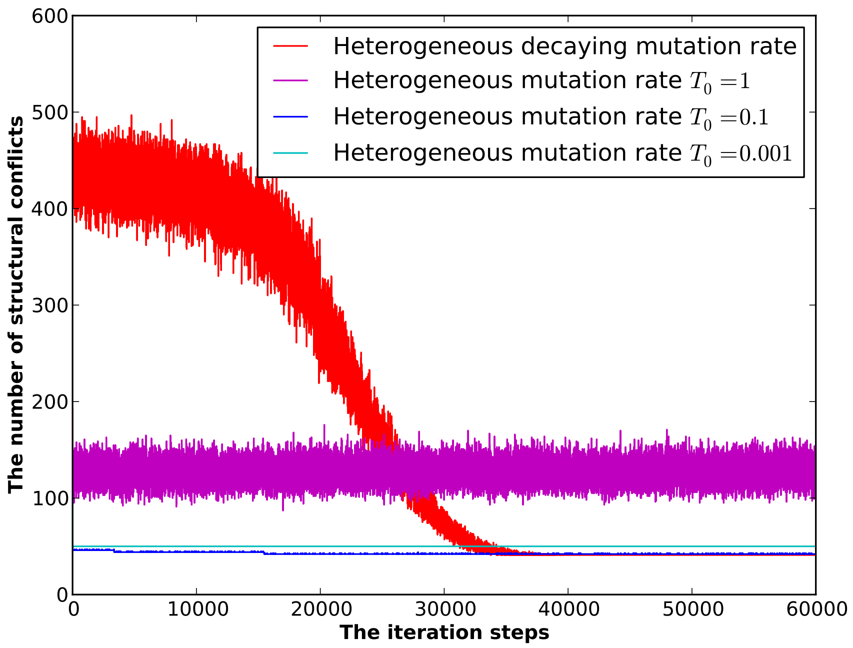

Fig. S2. Comparison of the evolutionary trajectories of the number of structural conflicts with different heterogeneous mutation rates and the heterogeneous decaying mutation rate. The initial noise parameter, decaying exponential, and damping period of the heterogeneous mutation rate are $T_0 = 100$, $\alpha = 0.9$, and $K = 100$, respectively.

trajectories of the network fitness with different parameter assignments. It can be found that the parameters can affect the convergence pace and state of the evolutionary process. Luckily, mutation happens on each node and thus the assignments of the mutation parameters are irrespective with the network size. That is, proper assignments of mutation parameters can be applied to various kinds of networks.

References

[1] S. Tan and J. Lü "Characterizing the effect of population heterogeneity on evolutionary dynamics on complex networks," *Sci. Rep.*, vol. 4, art. no. 5034, 2014

[2] S. Tan, J. Lü, X. Yu, and D. Hill, "Evolution and maintenance of cooperation via inheritance of neighborhood relationship," *Chin. Sci. Bull.*, vol. 58, pp. 3491–3498, 2013.

[3] S. Tan, J. Lü, and D. Hill, "Towards a theoretical framework for analysis and intervention of random drift on general networks," *IEEE Trans. Autom. Contr.*, doi: 10.1109/ TAC.2014.2329235, 2014.

[4] S. Tan, S. Feng, P. Wang, and Y. Chen, "Strategy selection in evolutionary game dynamics on group interaction networks," *Bull. Math. Biol.*, doi: 10.1007/s11538-014-0031-y, 2014.

[5] S. Kirkpatrick, C. D. Gelatt, and M. P. Vecchi, "Optimization by simulated annealing," *Science*, vol. 220, pp. 671-680, 1983.

[6] R. Milo, S. Shen-Orr, S. Itzkovitz, N. Kashtan, D. Chklovskii, and U. Alon, "Network motifs: Simple building blocks of complex networks," *Science*, vol. 298, pp. 824-827, 2002.

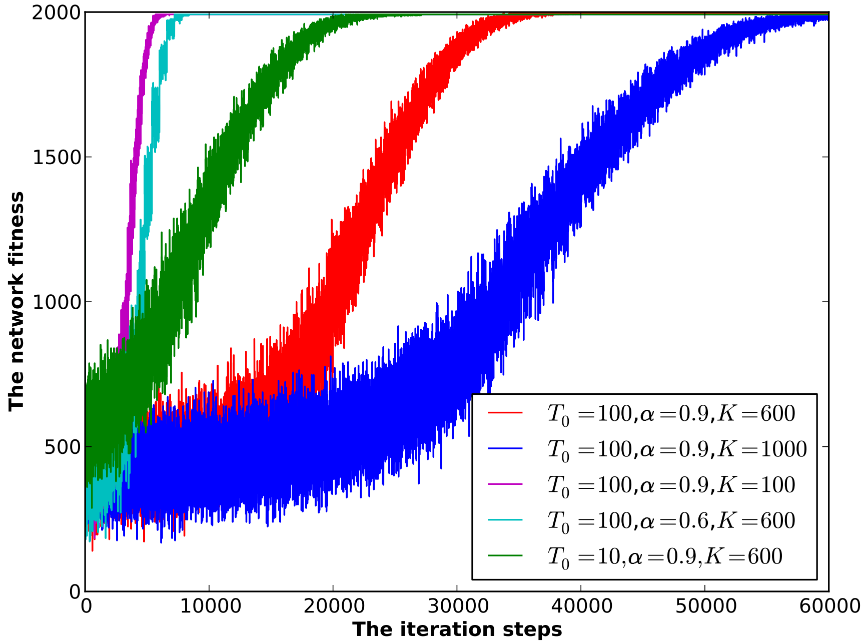

Fig. S3. Comparison of the evolutionary trajectories of network fitness with different initial noise parameter, decaying exponential, and damping period.
